# A valuation based theory of learning’s origin and development

**DOI:** 10.3389/fnsys.2025.1649748

**Published:** 2025-09-25

**Authors:** Vincent B. Moneymaker

**Affiliations:** Self-Employed, Brea, CA, United States

**Keywords:** learning, sleep, emotions, information valuation, dreaming

## Abstract

This paper proposes that learning in animals occurs thru sleep and is fundamentally driven by dynamic information valuation processes. These take the form of either pain and pleasure sensations or the more nuanced emotions that evolved from them. Acting as value identifiers, these sensations and emotions enable animals, from the simplest to the most complex, to mark valuable experiences for both retention and later recall. In this way, the paper argues that learning itself is made possible. The remainder of the paper explores the cognitive, neurological and behavioral implications of this framework, including several novel, testable hypotheses derived from it.

## Introduction

The fundamental sensations of pain and pleasure evolved as the most basic dynamic information valuation mechanism in the earliest animals with neuron-like structures. Emotions then evolved from pain and pleasure as a more nuanced valuation mechanism in animals possessing specialized emotion-generating structures. The purpose of these related information valuation mechanisms is to mark important memories for retention during sleep so that learning can occur. As such, the base feelings of pain and pleasure should be viewed as cognitively dependent sensations (just as emotions are) rather than as physical sensations. Which means they will be present in any species capable of learning, including species lacking neurons but possessing neuron-like structures, like sponges. Furthermore, all species capable of learning will be found to engage in some form of sleep because this is the process by which animals identify valuable memories in order to learn from them. As to emotions, it is submitted they are a ubiquitous feature of the brains of all higher-order animals, including mammals, birds, reptiles, and perhaps even fish and other classes of animals, such as cephalopods. The reason they’ll likely be found in all species possessing advanced cognitive structures, such as the amygdala, is they are the key neural development that enabled species to begin making more nuanced information valuations, which, in turn, led to the evolution of more complex cognitive abilities. Regarding the dreaming stage of sleep, it is hypothesized that it is critical to learning because it is the process by which newly stored memories are recalled so that their value can be accurately measured and assessed. As such, the purpose of dreaming is to refine learning that is the product of newly stored memories. Additionally, it is proposed that sleep cycles evolved to make the process of identifying and retaining valuable memories in service of learning more redundant and fault tolerant. So over multiple cycles, the brain examines new memories to ensure that informationally valuable ones are selected and preserved in as reliable a way as possible. It is further proposed that the number of sleep cycles an individual undergoes declines with age because progressively fewer short-term memories are sufficiently novel enough to merit being retained. As a result, fewer valuable memories results in less time being needed to transfer such memories to long-term storage. Finally, regarding the biological foundations of learning, neurons should be viewed as DNA’s information processing heirs. That’s because both mechanisms enable survival thru learning. However, in the case of neurons, they are self-contained heirs as they allow not only for the storage of information, but also its valuation, retention and recall. Most importantly, they enable dynamic learning on a daily and even hourly basis versus the much more static and comparatively inflexible mode of learning thru reproduction. This likely explains why the information processing capabilities of animals are unmatched by life in any other kingdom.

## The fundamental nature of learning

To explain the origin of learning, one must first define it. Learning is thus the ability to make information valuation determinations. And it is the product of three mechanisms working together, which are:

1. Storage
2. Information valuation
3. Selective retention and recall

So, in neural-based species, storage takes place via neurons or neuron-like structures. In turn, and as will be discussed hereafter, information valuation takes place via the sensations of pain and pleasure and, in more cognitively complex animals, emotions. Finally, the selective retention of valuable memories occurs via sleep, with pain and pleasure and emotions then guiding behavior once animals awake.

As to the importance of learning, it is one of the three principal capabilities animal species must possess in order to survive, with the other two being eating and procreating. We know how animals eat and procreate, but our understanding of how they learn is far from complete (Farrar et al., 2021). Of the three, learning is the most important because it effectively enables the other two. As a result, animals that are unable to learn over time have little to no chance of eating on their own, much less procreating, which guarantees they will not survive.

In proposing the within theory regarding how animals learn, this paper asserts that Albert Einstein’s principle of equivalence should be extended to biological processes (Begun, 2015). The point of this theoretical extension is that similar behaviors observed across a variety of species with a common genetic background will almost certainly be a product of the same underlying biological mechanisms.

Furthermore, the more widely a behavior or biological process appears in nature, the more fundamental it will be to the survival of species possessing it. As such, the equivalence principle should be used as a benchmark for determining how important particular behaviors or processes are to a species’ survival.

Viewing sleep thru this principle, its practically ubiquitous occurrence among animals points to it being as necessary to species’ survival as eating and procreating are (Cirelli and Tononi, 2008). So, given its fundamental nature, sleep, by process of elimination, is almost certainly involved in and responsible for learning. Which is the one crucial survival activity that still remains largely unexplained (Cirelli and Tononi, 2008).

In applying the principle of equivalence to animals, this paper proposes that all animals with neurons or neuron-like structures sleep because that is the process by which they learn. Specifically, and as highlighted before, this process involves animals using two different information valuation mechanisms to mark memories they generate while they’re awake. The first and more basic information valuation mechanism is that of pain and pleasure, which is present in all animals that are able to learn. The second, later evolved mechanism is that of emotions, which are present in animals with more complex neural frameworks that possess specialized emotion-generating structures, such as the amygdala.

During sleep, the strength of memories’ sensory and emotion tags signal to an animal’s learning network (be it a brain in some cases, a nerve net in others, or neuron-like structures in the oldest evolved creatures) as to which memories to keep and which to disregard. Then, when an animal awakes, the process begins again. The memory portion of its neural network, of which some part has been reset during sleep, is freed up to take in new sensory and emotion-marked memories. As part of a feedback loop, previously stored high-value memories are then recalled in order to guide behavior while also aiding in the generation of new memories. The result is animals are able to learn via neurons or neuron-like structures in a continuously adaptive way.

Ultimately, this is how animals store information that is important for their survival but is too complex or variable to be inherited by genetic means. So DNA functions as an information storage system but at the species level, encoding adaptive strategies through reproduction, mutation, and recombination across generations. This provides stability but is limited by the slow pace of evolutionary change.

Neurons, by contrast, represent an innovation that liberates information storage from generational turnover. So neurons enable the rapid, reversible, and context-dependent encoding of experience at a moment-to-moment level in individuals. In this way, neurons provide a qualitatively new substrate for learning that permits real-time adaptation to dynamic environments, which is a task DNA alone could never perform.

So, simplifying neural functioning to its essence, neurons’ ability to store information as valued by pain and pleasure and emotions and as selected thru sleep is how all animals with neurons or neuron-like structures are able to learn.

## Falsifiability

The theory presented here for how information valuation enables learning thru sleep sets forth a number of ways in which it may be tested and falsified. So, if the theory and its related explanations are correct, the following predictions should prove true.

a.    Memories will ultimately be found to be marked with pain and pleasure tags and emotion tags that signal their informational value.
b.    Once identified, memories’ pain and pleasure and emotion tags will be found to be the key component that determines whether memories are retained or discarded during sleep.
c.    Pain and pleasure and emotion tags that are present in human memories will be able to be traced back evolutionarily in other species, which will provide a record of how and when such tags evolved, particularly with respect to emotions and the exact extent to which specific ones are present in other species
d.    Sleep in species with bifurcated memory, i.e., with distinct short-term and long-term memory systems, will be found to include three identifiable functional stages: (1) memory value identification (likely associated with early NREM), (2) memory transfer and retention (linked to slow-wave sleep), and (3) memory assessment (occurring during REM/dreaming sleep). These stages will correlate with the prioritization and retention of valuable memories as denoted by the strength of their sensory and/or emotion tags and their disruption will impair learning outcomes.
e.    Dreaming will be found to occur in particular sensory modes based on the dominant sense a species utilizes while awake.
f.    Artificial intelligence systems will not be able to learn on their own until mechanisms are developed that allow them to independently value information and then test their information valuation determinations for validity thru the mechanism of choice.

## The mental trade-off justifying the inherent danger of sleep

Sleep’s fundamental role in learning explains why this seemingly maladaptive behavior is seen in practically all animals, even though its associated state of external unconsciousness (or lethargy in the case of animals with simpler neural networks) subjects its practitioners to extended periods of easy predation. Indeed, given that sleep essentially knocks animals out, often for hours at a time, it is safe to say that sleep is the single most dangerous activity animals engage in over the course of their lives. And yet animals do it day after day from the moment they’re born to the day they die.

They do so because that’s the trade-off that must be endured for animals to learn. For only during sleep’s enforced state of external unconsciousness can animals’ learning networks pause the generation of new memories so they can begin processing the day’s existing set of short-term memories. The end result is that during sleep, animals learn by keeping valuable memories while disregarding those with weak valuation tags.

As such, the theory that animals sleep in order to learn provides the most logical explanation for not only why animals sleep but also why it occurs in every animal that is able to learn. Alternate hypotheses for why animals sleep, such as engaging in energy allocation (Schmidt and Schindler, 2024), servicing a body’s essential metabolic functions (Sharma and Kavuru, 2010), or enabling an animal to better adapt to the Earth’s diurnal cycle (Freiberg, 2020), do not adequately explain why falling into a state of external unconsciousness is a necessary condition for these activities to occur. Simply put, each of these suggested reasons for sleeping could be carried out just as easily while animals are awake, and, thus, without having to render themselves uniquely vulnerable to predation.

In contrast, if animals are sleeping to learn, this would explain the need for them to enter into a state of unconsciousness when they sleep. That’s because new memory generation must stop for valuable short-term memories to be identified and retained. However, a significant argument against sleep’s purpose of enabling animals to learn is that other biological processes beyond neurological ones are thought to occur while animals sleep (Stich et al., 2021). As such, if sleep is intended to enable animals to benefit from processing valuable short-term memories into long-term ones so they can learn from them, then logic would dictate that only processes relating to this fundamental neurological activity should occur during sleep. But we know this is not the case, as bodily-related functions also occur during sleep (Dattilo et al., 2011).

The answer to this quite valid objection is that just as sleep, among other things, enables an animal‘s learning network to clear its short-term memories so it can take in new memories when it awakes, it seems logical that during sleep, an animal’s entire body would be rejuvenating itself for the same purpose. So, the therapeutic effects often seen in animals’ bodies while they sleep are likely done to prime animals’ sensory input systems, along with other related systems (perhaps down to a cellular level), for when they awake.

The end result is that when animals wake up, both their brains and their bodies are at the highest state of readiness for taking in new memories, which they can then learn from once they go back to sleep.

As to defining sleep, it’s proposed herein that sleep fundamentally involves the periodic suspension of sensory engagement with the environment in order to halt the intake of new information so that stored information can be evaluated for value. This valuation process enables the retention of high-value memories and the discarding of low-value ones. This is done to allow for learning on the one hand and to preserve storage capacity on the other.

Conventional definitions of sleep emphasize descriptive characteristics, such as reduced responsiveness to external stimuli, rapid reversibility, and homeostatic regulation (sleep rebound after deprivation). From the perspective advanced here, these features are not the essence of sleep but its observable correlates. They arise because the brain (or nerve nets or even neuron-like structures in simpler species) must minimize environmental input to carry out the valuation process. In other words, the existing criteria are best understood as external signatures of the deeper functional purpose of sleep, which is the processing of stored information for survival.

From this perspective, sleep is not an optional behavior but a necessary one in neural-based species. Thus, any species that possesses neurons or neuron-like structures should exhibit periodic sensory shutdowns consistent with sleep. It is asserted this will be the case because such shutdowns are the only way stored information can be effectively evaluated and managed for survival. Evidence that sleep-like states occur even in cnidarians, hydras and possibly sponges provides at least some support for this view (Flensburg et al., 2022; Kanaya et al., 2020; Keene and Duboue, 2018).

## The dynamic information valuation mechanisms of pain and pleasure are likely as ubiquitous as sleep is in supporting neural-based learning

Supporting the theory that sleep is ubiquitous in neural-based animals because it is the process by which they learn are studies showing that sleep occurs even in animals lacking complex brains, such as jellyfish and hydras (Kanaya et al., 2020; Keene and Duboue, 2018). Bolstering this theory are studies showing that animals such as jellyfish are also able to learn (Bielecki et al., 2023). And given the presence of neuron-like structures in sponges, which may be the earliest form of neural-based life, it seems likely that every species possessing neurons or neuron-like structures engages in some form of sleep while also possessing the ability to learn (Musser et al., 2019).

With more and more scientific data slowly eroding the belief that sleep only occurs in animals with complex brains (Nath et al., 2017), this paper proposes that once animals’ memory-based sensory and emotion tags are identified, it will be seen that these information valuation mechanisms are just as ubiquitous in the animal kingdom as sleep is. As such, they will be identified not just in animals with complex brains, but in the case of sensory-based pain and pleasure tags, they will be found in all animals, including those with rudimentary brains, and even in those possessing nerve nets and neuron-like structures. This is because these memory tagging mechanisms are a fundamental part of how all animals learn.

As to the nature of the sensations of pain and pleasure and the possibility of their existing even in animals with rudimentary brains or even simpler nerve nets or neuron-like structures, it is proposed that such sensations are cognitive-based and thus are consciously felt, even at a quite primitive level, in all animals that are capable of learning. It is further asserted that these sensations have to exist because no other mechanism is available for such species to assign value to their memories. And without such a valuation mechanism, learning can’t take place because learning is fundamentally about making information valuation determinations.

So, regarding how even primitive species such as sponges could be considered conscious, it is proposed that consciousness arises from the ability to perceive multiple frames of reference simultaneously. Which means that even at the level of individual neurons, or at the even more primitive level of neuron-like structures such as those found in sponges, these cellular components could possess a form of consciousness. This would be the case if they had an awareness of the present, such as, for example, in the form of environmental inputs, while simultaneously being aware of the past, such as in the form of neurally stored sensory information. As such, it is proposed that consciousness could arise even in individual neurons or neuron-like structures.

With pain and pleasure enabling a primitive form of awareness in the earliest evolved neural-based species, it is asserted that evolution soon selected for improved methods of information valuation and awareness. These came in the form of emotions, which, as previously noted, likely evolved from the base sensations of pain and pleasure.

As a second information valuation mechanism, emotions are also obviously cognitive-based. That’s because they are not bodily mechanisms like muscles or glands, but are produced by neural circuits in the brain.

Specifically, affective neuroscience has shown that emotions depend on dedicated neural systems such as the amygdala, prefrontal cortex, and insula (Damasio, 1994; LeDoux, 2000; Panksepp, 1998). Furthermore, they facilitate a wide variety of cognitive capabilities, including those of mental focus, decision-making, and memory encoding (Damasio, 2008; LeDoux, 2000; Panksepp, 1998). Empirical studies also demonstrate that experiences involving heightened emotional reactions are more vividly encoded and consolidated than neutral ones, underscoring emotions’ role as a higher-order information valuation system (Kensinger, 2009).

So due to emotions being more informationally nuanced versions of pain and pleasure, they require more complex neural structures with greater processing power to generate and utilize them. As such, they will be found in all mammals and birds. And, likely, they will also be found in most reptiles, fish, and other species with higher-order brains, such as squids and octopuses. Simply put, it is proposed they’ll be found in any species that possess amygdalas or amygdala-like cognitive structures, which are responsible for generating the emotion tags that are hypothesized to assign value to memories.

As to the term “emotion,” it is submitted this is the correct usage to apply to all such memory markers, no matter the species possessing them, because they are mental feelings that allow animals to consciously assess the relative importance of their interactions with their environment. A potential objection to this assertion is that to ascribe such mental feelings to other animals is to engage in anthropomorphism. However, the concept of anthropomorphism may itself be anthropomorphic, given that it ascribes a uniqueness and, indeed, a superiority to human biological processes that simply does not exist.

So, just as humans share DNA with all living animals, it is submitted that humans share the same basic sensations of pain and pleasure, albeit often in less cognitively aware forms, with all other animals, and emotions with a great many as well (Smithsonian National Museum of Natural History, 2024). Once this is shown, it will demonstrate that humans share essentially the same biological mechanisms for learning that every other learning-based species possesses. And the proof for this assertion will be found in the identification of our memories’ respective sensory and emotion tags, followed by their tracing back to species with earlier evolved versions.

## How the information valuation mechanism of pain and pleasure first evolved to enable neural-based learning

It is proposed that all life requires some form of information valuation in order to survive. The most basic form of information valuation occurs through the selection of genetic information via reproduction and natural selection. But the problem with this form of information valuation is it essentially remains static until an organism reproduces. To respond to this challenge, species evolved dynamic information valuation mechanisms that enabled information’s value to be updated much more quickly, even to the extent of it being valued on a moment to moment basis.

As such, it is submitted that the first dynamic valuation mechanism came in the form of microorganisms’ development of proto-immune-like responses to outside attackers. Specifically, this first dynamic mechanism likely evolved to enable microorganisms to distinguish between helpful foreign particles like food and harmful ones like pathogens (Janeway et al., 2001). Thus, proto-immune-like systems formed life’s first dynamic learning system, albeit at a cellular level (Netea et al., 2011).

As for life’s first consciousness-based information valuation mechanism, which collectively are the base sensations of pain and pleasure, it is proposed that this mechanism was developed by re-purposing certain capacities of life’s earlier evolved learning system, otherwise known as the innate immune system (Martins et al., 2023).

In this regard, it has been persuasively theorized that neural information processing likely began with a combined sensory-neuro-type structure (Mackie, 1970). This type of structure started as a sensor mechanism that then triggered an action. Then, a proto-neural part was added to the cellular structure. This enabled sensory-generated information to be taken in and transmitted to and stored in the proto-neural part of the structure. Upon such information being recognized and stored, the pre-existing action part of this structure then triggered a response. So these proto-neurons were able to induce actions in an organism without requiring a central nervous system to function.

For example, one form of these proto-neurons could have evolved to detect digestible carbon-based particles, with an action to consume such particles then being triggered. These initial proto-neurons might have been followed by others that could identify non-carbon-based particles, which, upon detection, resulted in the proto-neurons triggering an action to expel such particles.

Thereafter, it is proposed that evolution repurposed several aspects of organisms’ innate immune systems to enhance the information-processing power of these proto-neurons. First and most critically, it is submitted that this information processing system required a new information valuation mechanism to function. As such, evolution likely repurposed the closest available equivalent, which was the innate immune system’s valuation mechanism for distinguishing between an organism’s own cellular components and foreign invaders (Anaya et al., 2013).

So if an action a proto-neuron triggered resulted in the organism’s health deteriorating, that information could be transmitted back to the proto-neuron to be stored along with the information that had initially triggered the action. With proto-neurons now receiving information valuations on their actions, which likely evolved quite quickly into sensations of pain and pleasure, they could begin learning similarly to how the innate immune system was already doing.

After that inflection point, evolution could have quickly selected for one-way information transmission pathways to evolve into two-way pathways. So, with immune-like components developing chemical pathways to transmit information to the first neuron-like cells, they could then begin receiving information back thru these same chemical channels. Once that happened, the floodgates would have opened for proto-neurons to start transmitting information to each other.

From there, the next step would have been the evolution of a proto-nervous system. This would have allowed triggering signals from specialized information-processing proto-neurons to be transmitted at a distance to specialized action-triggering proto-neurons. In turn, this would have enabled organisms to begin developing extended multi-cellular body plans.

A relatively simple example of how such a proto-nervous system might have evolved would be for a set of proto-neurons that had detected a nearby heat source to send signals via repurposed immune-like chemical transmitters (such as cytokines) to other neurons that had specialized in triggering an organism to move. So, one set of heat-detecting proto-neurons could have used the proto-immune system’s chemical pathways to send signals to another set of proto-neurons to begin moving away from the potentially harmful heat source.

It is submitted that after a proto-neural information storage mechanism had been developed, along with a mechanism to assign value to such information, certain aspects of the innate immune system were repurposed a third time, leading to the resetting of proto-neurons. So, in the case of proto-neurons with sufficiently weak pain/pleasure valuations, they would have been identified and then reset in order to take in new information. This is how sleep would have first evolved. And, as noted before, its purpose would have been to shut off the intake of new sensory information so that proto-neurons and, later on, actual neurons could be made ready to store new and potentially more valuable information.

With proto-immune system functions being repurposed to aid in the evolution of neurons, nervous systems, and, ultimately, brains, this would explain why various cellular components and chemical transmitters, such as cytokines, complement proteins, and certain types of glial cells, such as phagocytes, astrocytes, oligodendrocytes, radial glia, and Schwann cells, have significant roles in the functioning of both immune systems and neurological systems (Dantzer, 2018).

## Pain and pleasure as the foundational valuation mechansim for neural learning

As to the base sensations of pain and pleasure, this paper asserts that the entire reason they were evolutionarily selected for is to provide value determinations on information. Specifically, they evolved to provide value determinations as to which sensory-derived information should be immediately acted upon via cognitive-based structures and then retained for learning purposes.

So painful and pleasurable reactions to sensory information serve as indirect markers for the presence of neurons or neuron-like structures in animals and even in the earliest evolved species. Furthermore, as the first valuation mechanism for cognitively stored information, pain and pleasure are, as noted before, consciously dependent sensations rather than physical sensations. Which explains why reactions to painful interactions, even in the earliest evolved animal species, are tempered when analgesics are applied, or eliminated outright in the case of anesthetics (Maldonado and Miralto, 1982; Morgan et al., 2007). As such, this paper proposes that pain and pleasure’s biological components will be found in all animals with cognitive-based learning networks, from the most primitive to the most complex.

Regarding their scope, pain and pleasure evolved so that no matter what environment an animal encountered, the sensory information derived therefrom could be interpreted via a single metric. Whether the interaction involved environmental conditions (such as in the case of heat, cold, acidity, or salinity) or an environmental encounter resulting in, for example, a successful mating or a nearly fatal attack, all could be measured on a continuum thru the yardstick of pain and pleasure.

And in serving as valuation tags or markers for sensory information that was being cognitively stored, pain and pleasure could themselves begin triggering reactions. So, a pain valuation would indicate to a proto-neuron that the action it had initiated was harmful, thereby resulting in its stopping the action. Or it might trigger a contrary reaction, such as by signaling to an organism to move away from whatever was causing the feeling of pain. In contrast, a pleasure tag would indicate an action was helpful, so the proto-neuron should continue triggering the action. Such a system would not only enable organisms to immediately respond to environmental cues, but also enable them to learn from sensory-derived information. As such, this is how a new learning system could have evolved without neurons or a nervous system initially being present.

As an example of this, if a relatively simple multi-cellular organism, such as a sponge, just ate, the sense of pleasure derived from that activity would be used to mark the sensory information associated with the event – in other words, the ‘what’, ‘when’ and ‘where’ of the event. This pleasure-tagged information would then get stored in neuron precursors, such as a sponge’s neuroid cells (Marshall, 2021). Once multi-cellular organisms were able to recall such information, for example thru new sensory data triggering the same proto-neuron into action, even relatively simple animals, such as sponges, could start learning.

Using pain and pleasure to mark memories for assessment could then potentially explain several curiosities seen in animal biology and behavior. The first of these curiosities is biology-based. It involves finding neuron-like structures, that are associated with digestion, in sponges, which lack both neurons and a nervous system (Musser et al., 2021). It is proposed that such structures may have evolved in order to detect when a sponge has fed, thereby enabling the information associated with that critically important behavior to be immediately stored. This would, in turn, allow for learning to take place based on the pleasure-marking of the information.

Relatedly, this might also explain why all animals with a central nervous system possess a “second brain” in their digestive systems, which is otherwise known as the enteric nervous system (Furness and Stebbing, 2017). This second or, in reality, “first” brain (given that it evolved before the central nervous system) was likely selected for because it enabled animals’ learning networks to exercise a great degree of control over when and what they ate.

Another curiosity associated with the use of pain and pleasure as a valuation mechanism is behavior-based. Specifically, it involves the behavior of feeling sleepy after eating a big meal that many animals, including humans, demonstrate (Gallagher and You, 2014). This could be a valuable information processing tactic descended from when primitive animals first began using the base feelings of pleasure and pain to mark memories for retention. So animals get sleepy after enjoying a big meal because their learning networks recognize, based on the pleasure-tagging of the memories associated with this activity, that something highly informationally valuable has just occurred. As a result, a feeling of sleepiness is triggered. After an animal goes to sleep, its brain is then able to rapidly capture the valuable information that led to the big meal and learn from it.

Similarly, when males of different species have sex, there is anecdotal evidence that they tend to feel tired afterward. This phenomenon has been so widely observed, particularly as it relates to humans, that it is often remarked upon in humorous articles on the topic (Moy, 2022). So, in the case of after-sex, the brain triggers a feeling of sleepiness in males to capture the information associated with the evolutionarily critical activity of reproduction. And brains, and even simpler neural networks, may have been engaging in these sorts of sleep-based information-capturing activities since animals first evolved consciousness-based learning networks.

A key question presented though is if pain and pleasure serve as the first valuation mechanism that enabled learning to occur in neural-based species, how could early evolved species possessing simple neural structures have experienced the sensations of pain and pleasure? For example, how could jellyfish, hydras, or the even earlier evolved phylum of sponges, which don’t possess neurons at all, have experienced these seemingly complex feelings?

An important clue is found in the neural chemistry of these ancient species. So, the very neurotransmitters that underpin pain, pleasure, and reward in mammals, i.e., serotonin, dopamine, GABA, and glutamate, among others, are also present in cnidarians and potentially even in sponges’ neuroid cells (Elliott and Leys, 2010; Kass-Simon and Pierobon, 2007; Musser et al., 2019). Furthermore, in these comparatively simple animals, neurotransmitters appear to trigger functional effects, such as contractions and feeding modulation, which in higher animals correspond to pain/pleasure valuations (Musser et al., 2019). One specific example is telling, which is that GABA and glutamate trigger contractions in sponges (Musser et al., 2019; Flensburg et al., 2022). The evolutionary conservation of these neurochemical messengers thus strongly suggests that the neurotransmitter-based valuation of information was present in animal species long before specialized cortical circuits evolved.

So, if the conventional view is accepted that neurons evolved to store and recall information for purposes of survival (Moroz and Romanova, 2022), then primitive neurons would have only needed basic valuation signals to mark valuable memories for retention and recall. It’s submitted this is essentially why the same set of neurotransmitters that trigger pain/pleasure signals in complex species, such as mammals and avians, are also found in ancient species such as jellyfish and sponges (Liebeskind et al., 2017).

As a result, even in these ancient species, neurochemical-based sensations of pain and pleasure appear to be present in some form. This strongly suggests they help to guide future behavior based on remembered information that has been previously stored, which is the essence of learning.

Evidence that jellyfish and hydra display sleep-like states further supports this proposition (Kanaya et al., 2020; Keene and Duboue, 2018). This is so because the presence of sleep states is precisely what should be expected if these ancient species are valuing information, which in turn periodically requires sensory intake to be paused so that valuable information can be identified and retained, while low-value information is discarded.

Lastly, the biology of jellyfish provides direct support for the assertion that ancient neural-based species use pain and pleasure to value information. Specifically, cnidarian stingers weaponize pain for both predation and defense, including against other jellyfish (Jouiaei et al., 2015; Schlesinger et al., 2008). It is difficult to see why evolution would select for such a pain-based weapon unless cnidarians themselves could register pain as an aversive valuation signal.

So taken together, the presence of conserved neurotransmitters, rudimentary memory, sleep-like states, and the use of pain in predation/defense all strongly support the hypothesis that even the earliest neural-based species employed pain and pleasure as the first information valuation mechanism so that neural-based learning could occur.

As a result, the sensations of pain and pleasure should be thought of as a single information valuation mechanism which evolution selected for in the earliest cognitive-based species to distinguish between useful and useless information and thereby make neural-based learning possible.

## Emotions as neural learning’s more nuanced information value mechanism

Emotions evolved from the first cognitive-based valuation mechanism of pain and pleasure in order to provide more nuanced valuations for information stored in neurons. As such, cognitive-based systems used the same memory tagging mechanism for emotions as was being used for pain/pleasure valuations. And, as with pain and pleasure, emotion tags allowed brains to determine whether a memory, based on the strength of these tags, was sufficiently valuable that it should be kept during sleep. The same mechanism then allowed memories with relevant emotion tags to be recalled while an animal was awake.

So, just as evolution’s information processing imperative utilized the foundation of proto-immune signaling to bootstrap pain and pleasure as a valuation mechanism, pain and pleasure were then used, in turn, to bootstrap the evolution of a second, more potent mechanism for evaluating information. Emotions should thus be viewed as higher-level flavors or echoes of the base cognitive sensations of pain and pleasure (Kringelbach and Berridge, 2018). This almost certainly explains why they often produce unpleasant physical sensations in the case of negative emotions and pleasant physical sensations in the case of positive ones. This is so because, as the valuational heirs of pain and pleasure, emotions almost certainly have similar underlying neurobiological compositions, but more on this connection later (Chaudhry and Gossman, 2025; Li et al., 2025).

Regarding the need for emotions, they provided valuation assessments for memories to enable learning but in much more effective ways. And indeed, they were likely a critical factor in the development of complex capabilities such as brooding and child-rearing, as will also be discussed later (de Waal, 2008). As to how they enabled more complex learning capabilities, they did so first by allowing species to consider their reactions to environmental actions at a much more conscious level than at the nearly automatic level typically triggered by pleasant and, particularly, painful sensations and associations.

Second, with consciousness having evolved in species to give a greater, more multi-faceted sense of the present, utilizing emotions as information valuation tags enabled species to avoid regurgitating memorialized sensations of pain or pleasure. Such sensations likely proved problematic at a conscious level because they might not only overwhelm an animal’s ability to generate informationally comprehensive new memories, but they might also sidetrack animals from effectively responding to whatever dangers or potentially rewarding activities they were facing at any particular moment in time.

Finally, and perhaps most importantly, emotions enabled species to respond in more nuanced ways to environmental interactions than the blunt sensations of pain and pleasure were capable of. So memories could be stored with a variety of emotion tags and even combinations of them, which would then help animals to respond to external events in much more adaptively appropriate ways. This would be in contrast to the more instantaneous “fight or flight” response a pain-marked memory might trigger or the nearly automatic behavioral response of wanting “more” in the case of pleasure-marked memories (Glazer et al., 2018; Vila et al., 2007).

As an example, a memory marked with a negative emotion might produce a feeling of anger in some cases or fear or revulsion in others. In each case, a completely different yet adaptively appropriate response would be the result. Or, a memory marked with a positive emotion might result in a mental feeling of happiness or satisfaction in some cases or curiosity in another depending on the environmental cues triggering the memory’s recall. The actions resulting from these positive emotions would then be completely different based not only on the emotion but on the context of their recall.

Accordingly, the adaptive power of emotions as the second evolved mechanism for evaluating memories is obvious. Different emotions, though ultimately based on the fundamental sensations of pain and pleasure, enabled animals to react in entirely different ways depending on what was the most adaptively suitable response to the particular circumstances they found themselves in.

## Empathy as an evolutionary step enabling social and developmental learning

It seems likely that the evolution of the emotion of empathy, along with its sister emotions of compassion, sympathy, and pity, led to a dramatic change in species’ behavior, as well as in species’ cognitive abilities (Masserman et al., 1964). As to why this occurred, it is theorized that empathy, compassion, sympathy, pity, and other related emotions are the first complex emotions to have evolved among animal species (Panksepp and Panksepp, 2013).

What makes such emotions complex is they are essentially double-layered, which means the mental feelings they produce are based on how one thinks another is feeling. So, in effect, these complex emotions produce two levels of emotions. One that is felt by the empathizer and another that is perceived as being felt by the subject of the empathetic feeling.

As to the change these emotions promoted in species’ behavior, it appears that this expansion of species’ emotional toolkits led to animals beginning to care for their young (de Waal, 2008). Which, in turn, became a significant driver of brain expansion, likely due to child-rearing being one of the most complex activities animals engage in (van Schaik et al., 2023). This is the case because child-rearing is one of the few animal behaviors that extends out over weeks, months, and sometimes even years while simultaneously requiring animals to provide for themselves and their children, as well as in having to act to protect both themselves and their children.

With the evolution of complex emotions leading to offspring-protecting and child-rearing behaviors, this necessarily resulted in animals becoming concerned, for the first time, with the survival of others. Those others would have started off with their children, then their mates, and then, subsequently, even their kin. This behavioral change would have been in stark contrast with the behavior of animals who remained focused solely on their own survival.

The evolution of emotions concerned with the wellbeing of others thus propelled animals into the second great leap in consciousness. The initial leap occurred when animals’ neural structures made them aware of their environments. The second leap then occurred when empathetic emotions opened animals’ eyes to the difference between themselves and others. The end result was that the evolution of complex emotions led to animals becoming self-aware.

## Complex emotions as drivers of problem-solving and adaptive learning

It is asserted that when the lineage of our emotions is tracked backward in evolutionary history, it will be found that the “motherly” emotions such as empathy, affection, love, protectiveness, tenderness, and worry developed in the first child-protecting species. Specifically, it is proposed that the evolution of such “motherly” emotions likely occurred around the time species began protecting their eggs after they were laid. From there, yet further emotions evolved relating to the guarding and nurturing of children, leading to species beginning to protect and teach their children after they hatched or were born. So, it is hypothesized that the development of the first “motherly” emotions is actually what led to these child-protecting behaviors. And at each stage, species’ brains expanded to help animals successfully act upon these emotions that were, in effect, encouraging their nurturing behaviors (Edgar et al., 2011).

With the expansion of brains being propelled by the complex behaviors required to successfully protect and then rear animals’ offspring, it is asserted that this led to the third leap in consciousness observed in animals, which is problem-solving behavior. More precisely, what the third leap in consciousness allowed animals to do is create new ideas. And in doing so, what was actually happening in animals’ minds is that they were projecting themselves into the future to anticipate the results of their new ideas.

This is how the imagination first evolved. So, once an animal developed a new idea that led to a successful problem-solving behavior, the behavior could then be taught to others. As such, in species possessing “motherly” emotions, we’ll likely find that many of them possess the ability to problem-solve.

Specifically, we’ll find complex “motherly” emotions in all mammals and birds, likely in crocodiles and even in some species of snakes such as cobras and pythons and other reptiles such as monitor lizards. And, finally, we’ll possibly even find them in some species of fish. Once we’re able to identify the genes associated with the development of such emotions, we may even find them in some species of dinosaurs that cared for their young, assuming, of course, that we’re ultimately able to find the DNA for a wide variety of dinosaur species.

Thus, it is proposed that empathetic emotions came first in one or more species, which led to the guarding and nurturing of the eggs they laid. This, in turn, led to bigger brains, which evolved to carry out the more complex activities associated with caring for one’s young (van Schaik et al., 2023). And as child protection and rearing become even more involved, and with such activities taking place over progressively longer periods, this led to the progression in brain size that we see in many species, along with a cognitive leap in consciousness as evidenced by problem-solving behaviors (Uomini et al., 2020).

And with brain size generally increasing in proportion to how much time and effort a species puts into caring for their young (Barton and Capellini, 2011), we will likely find in the future that some species with comparatively large brain sizes, such as, for example, certain species of sharks, that are generally presumed not to care for their young, actually do care for them in some fashion. And in such species, problem-solving behaviors will also likely be evident.

## How the valuation mechanisms of pain and pleasure and emotions relate to each other

Pain and pleasure represent the most fundamental valuation mechanism in neural systems. They operate at the level of immediate survival by marking experiences as either harmful to avoid or beneficial to repeat. These signals are basic, fast, and essentially universal among species possessing neurons. As such, they provide the first layer of information valuation that signals which experiences should be retained and which discarded in order to guide learning.

Emotions, by contrast, are more complex and evolved later as a higher-order valuation system that requires much more sophisticated cognitive structures, such as the amygdala, to function. So, whereas pain and pleasure are binary in nature, signaling positive versus adverse outcomes, emotions provide a broader spectrum of nuance. As such, they integrate context, social meaning, and anticipated consequences, allowing experiences to be valued along multiple dimensions rather than just one. For example, fear does not merely register ‘pain’ but anticipates future threats. Empathy, on the other hand, extends valuation to the welfare of others.

Thus, in terms of hierarchy, pain and pleasure are the evolutionary source of emotions, thereby providing the functional foundation upon which emotions build. As a result, emotions serve to incorporate and extend the logic of pain and pleasure, embedding those primal signals within a more elaborate cognitive framework that enables flexible and socially adaptive learning. In this way, pain and pleasure remain the bedrock of information valuation, while emotions represent a more sophisticated, context-dependent system layered on top of that foundation.

## Dreaming as an extension of emotional valuation for learning across species

It is submitted that dreaming exists to improve learning. So dreaming occurs after new memories are woven into animals’ long-term memory frameworks during sleep. What dreaming does [which this paper suggests should be relabeled the “dreaming” stage of sleep rather than the rapid-eye movement (“REM”) stage of sleep] is ensure new memories are not only properly stored but that their informational relevance is properly measured and assessed for recall when an animal awakes.

This is why dreaming emulates wakefulness, with brain waves generally being unsynchronized, except as to the gamma wave oscillations seen during both dreaming and wakefulness (Hobson and Pace-Schott, 2002), as well why dreams typically have life-like narrative structures. Dreaming thus serves to trigger the recall of newly stored memories, just as might occur during wakefulness, but with the brain having much greater control over their manner of recall.

This process, it is submitted, is what enables the refinement of learning, thus explaining why disruptions to the dreaming stage of sleep can have a significant impact on the recall of physical as well as mental skills that have been newly learned (Rasch and Born, 2013). And with nearby, yet potentially unrelated, thoughts, ideas, and associations being activated during this process, this further explains why dreams can seem so real while at the same time being so disjointed.

The critical nature of both storing adaptively valuable information and ensuring its wakeful relevance thus points to the strong likelihood that many animals dream while they’re asleep (Louie and Wilson, 2001). Accordingly, it is proposed that dreaming occurs in many vertebrate species with REM or REM-like phases of sleep, which again this paper proposes should be relabeled as the “dreaming” stage of sleep. So, this will likely hold true for all mammals, birds, and most, if not all, reptiles, and possibly fish. It is also possible, and perhaps even likely, that dreaming may be occurring in invertebrate species like cephalopods.

## The neurological basis for learning through information valuation

If pain and pleasure and emotions act as information valuation mechanisms to enable learning, how do they do so at a neurological level? It is proposed they do so by being stored as markers or tags in memories. Specifically, they are hypothesized to be written into the presynaptic membranes of neurons when synaptic vesicles operate in kiss-and-run mode (Harata et al., 2006), with slow oscillation brain waves (Csercsa et al., 2010) in the range of 0.5–1.0 Hz likely being the signature of action potentials triggering this activity.

The manner in which they are asserted to be written into the presynaptic membrane is by vesicles docking at active zones (Südhof, 2012) on the presynaptic membrane. Lipid rafts (Hering et al., 2003) then act as transport mediums for transferring information to and from scaffolding protein complexes (Ackermann et al., 2015) located in the presynaptic membrane. As such, scaffolding protein complexes are hypothesized to be the ultimate site of memory storage, including that of the sensory and emotion tags that are asserted to be critical to the retention of memories.

But even if this hypothesis ultimately turns out to be incorrect, what is not subject to dispute is neurotransmitters are involved in the formation and signaling of both pain and pleasure and emotions (Chaudhry and Gossman, 2025; Li et al., 2025). Even more pertinently, there is a significant overlap in neurotransmitters involved in feelings of pleasure and positive emotions (such as dopamine, oxytocin, serotonin, endorphins, and norepinephrine) (Esch and Stefano, 2004), as well as in feelings of pain and negative emotions (e.g., glutamate and gamma-aminobutyric acid) (Elman and Borsook, 2018). It is submitted that this correlation is not accidental and should be viewed as significant evidence of an evolutionary association, with pain and pleasure’s neurological components having evolved into emotions once brains developed emotion-generating structures like the amygdala.

Lastly, and as previously noted, the fact that neurotransmitters associated with pleasure and pain are found even in ancient species such as sponges provides significant evidence for the hypothesis that animal species possessing only neuron-like structures, such as sponges, are nevertheless capable of feeling pain and pleasure at a basic level (Ellwanger et al., 2007). So if ancient animal species like sponges can feel pain and pleasure, the logical conclusion to draw from this is that pain and pleasure evolved as the first neural-based information valuation mechanism, which then led to the evolution of emotions.

## Sleep’s enabling of learning

It is submitted that sleep’s function is to enable learning. As discussed in prior sections, sleep does this at a basic level by shutting down the generation of new memories so that valuable short-term memories can be identified and transferred to long-term storage. Once an animal wakes, relevant memories not only serve to guide behavior but become the building blocks for skills that enable survival.

The stages of sleep demonstrate how this occurs. In the first stage of what is known as non-rapid eye movement (“NREM”) sleep, this is marked by a light transitional state that typically lasts just a few minutes (Patel et al., 2025). It is submitted that there are two purposes for this transitional state. The first is to allow the brain sufficient time to shut down sensory inputs that would otherwise result in the generation of new memories. The second is to prime certain parts of the brain for the next stage of NREM sleep, otherwise known as stage 2 of sleep. As to how this occurs, the thalamus is hypothesized to be primarily responsible for the process of shutting down sensory functions (such as seeing, hearing, and feeling). This is likely the case given that it is appears to be the central hub both thru which memories are generated and consciousness arises (Ward, 2011).

In stage 2 of NREM sleep (Patel et al., 2025), it is submitted that cognitive structures in the brain, particularly associated with the thalamus, begin marking valuable short-term memories for transfer to long-term storage. Sleep spindles (Mednick et al., 2013), in the approximate frequency range of 11–16 Hz, along with K-complexes (Happe et al., 2002), which contain dominant frequency components in the range of 0.5–4.0 Hz, are proposed to be the brain wave signatures associated with this activity. Specifically, it is asserted they are the aggregate signatures of action potentials triggering vesicles to mark valuable information for transfer that is stored in the presynaptic membranes of short-term memory neurons.

As this occurs, delta waves, in the frequency range of 1–4 Hz, are also seen during stage 2 of sleep, though not nearly at the level seen during stage 3 of NREM sleep (Patel et al., 2025; Prerau et al., 2015). It is proposed that these delta waves are the signature of action potentials triggering vesicles to operate in kiss-and-run mode. The purpose of this is to erase short-term memories with little to no value, principally as determined by the relative weakness of their sensory and/or emotion tags.

The presence of differing types of brain waves during particular stages of sleep thus raises the possibility that the stages of sleep are not as sequential as they might otherwise seem (Siclari et al., 2018). So it is hypothesized that the functions of each stage actually overlap on a consistent basis (Siclari et al., 2018). As such, while delta waves are normally seen during stage 3 of sleep, the fact that they can also occur during stage 2 of sleep should be viewed as evidence that the erasure of short-term memories is being carried out in both stages of sleep. So, memories having little to no informational value get discarded, likely during stage 2 of sleep. While highly valuable memories that have already been transferred to long-term memory don’t get erased until stage 3. The end result is short-term memory is cleared to take in new memories when a subject next wakes up.

During stage 3 of sleep, it is proposed that valuable short-term memories, as denoted by the marking they’ve undergone in stage 2 of sleep, are transferred to long-term memory. High-frequency oscillations in the range of 80–250 Hz, and otherwise known as “ripples,” are proposed to be the signature of this activity taking place (Frauscher et al., 2018). Specifically, what fast ripples signify is valuable short-term memories are being read via vesicles operating in collapse mode and then transmitted thru hippocampal-thalamic circuits to the thalamus (Aggleton et al., 2010). From the thalamus, they’re then transferred to the neocortex via thalamocortical circuits (George and Das, 2025).

Once these short-term memories reach the neocortex, they are written into long-term storage. The signature of this activity taking place is proposed to be slow-oscillation waves occurring in the 0.5–1 Hz range (Csercsa et al., 2010). Specifically, and as noted before, slow oscillations are the signature of action potentials triggering vesicles to write information via their kiss-and-run mode of operation to the presynaptic membranes of neurons in the neocortex.

As with stage 2 of NREM sleep, it is proposed that different functions also occur during stage 3 of sleep. In this case, dreams sometimes occur during stage 3 even though they are typically associated with the last stage of sleep (Siclari et al., 2018). As such. because dreaming overlaps between stage 3 of sleep, where little to no eye movement occurs, and the last stage of sleep that is typically defined by rapid eye movement, it is submitted that this stage of sleep is more properly viewed as the “dreaming” stage of sleep.

Regarding the “dreaming” stage of sleep, it is hypothesized that it has multiple functions. The first is akin to a quality control measure and is intended to ensure that short-term memories transferred to the neocortex have been properly written into long-term memory. So activating these memories in order to trigger their recall to the thalamus, as they would be during wakefulness, likely ensures they have been stored properly (Wolff and Vann, 2019).

The second reason for the “dreaming” stage of sleep is to enable the rehearsal of memories associated with newly learned physical and mental skills. So, replaying them during dreaming ensures that the sequencing and interconnection of the synapses storing such memories are both confirmed and reinforced. And this likely occurs not just in the neocortex but also in the nervous system. Specifically, it is proposed this occurs with respect to interneurons (Kepecs and Fishell, 2014) and motor neurons (Zayia and Tadi, 2025) that actually execute the commands that result in such skills being carried out.

The third reason for activating transferred memories is to ensure that the postsynaptic membranes associated with newly stored memories are able to properly assess their informational value for recall purposes. This is critical because if such memories are to be made useful, it is not enough that they get stored. Their informational significance and relevance also have to be measured. It is proposed this happens via postsynaptic membranes lowering or raising their gateways to allow information thru based on the value of the information stored in presynaptic membranes.

So, ultimately, what dreaming represents is the purposeful creation of a virtual mental environment that enables newly stored memories to be recalled in much the same way they might be during wakefulness. This process allows the informational context and value of new memories to be accurately “gated” in postsynaptic membranes, thus resulting in the direct refinement of learning. This is done during sleep, as opposed to during wakefulness, because the dreaming stage of sleep gives the brain much more control over how newly stored memories are activated and recalled. Furthermore, it is asserted that this is likely the most important part of sleep in relation to long-term potentiation (“LTP”) and long-term depression (“LTD”) (Bliss and Cooke, 2011)

It is submitted that sleep disruptions support this hypothesis. Specifically, sleep studies indicate that declarative memories benefit from stage 3 of NREM sleep. This correlation makes a great deal of sense if stage 3 is when short-term memories are being transferred to and written into the presynaptic membranes of neurons associated with long-term memory in the neocortex.

With respect to procedural memories, sleep studies indicate that their effective recall benefits significantly from the “dreaming” stage of sleep. This also makes sense if this is the stage of sleep where procedural memories get rehearsed as well as when their informational relevance is measured via gateways in the postsynaptic membrane (Caire et al., 2025).

And this would also explain why there is a rebound effect after patients stop using anti-depressants (Feriante and Singh, 2025). That’s because one of the side effects of anti-depressant usage is that dreaming stops (Feriante and Singh, 2025). So, with procedural memories being transferred to and stored in the neocortex during stage 3 of sleep, the necessary follow-on step of information assessment and gating doesn’t take place. Instead, this follow-on step has to wait until anti-depressant usage ends and dreaming starts up again (Feriante and Singh, 2025).

Additional support for the dreaming hypotheses presented in this paper can be seen in the curious lack of REM sleep seen in cetaceans (Lyamin et al., 2008). This has long been considered a mystery in sleep research because many other mammalian species do experience rapid eye movement during sleep. It is proposed that the reason for the discrepancy between cetaceans and other mammals is that cetaceans’ primary sense isn’t seeing but hearing.

So, if dreaming’s purpose is to refine valuable memories in order to enable learning, it seems likely that cetaceans’ most important memories will be aural in nature (Mooney et al., 2012). Whereas with humans and other land-based mammalian species, their most important memories will be visual in nature. As a result, humans and other eye-dominant species experience REM sleep because they are seeing their memories during sleep. Whereas whales and dolphins are likely hearing their memories during sleep.

Lastly, regarding the deleterious impact interruptions of stage 3 sleep have on bodily functions and overall health, it is asserted there is a straightforward explanation for this. Both the brain and the nervous system have mechanisms in place to clear the waste products resulting from action potentials firing in neurons throughout the brain and the body (Xie et al., 2013). So if stage 3 of sleep is interrupted, these clearing mechanisms cannot do their jobs. This is why disrupting stage 3 sleep not only has a negative impact on brain functioning but on the health of the body as well.

To this end, it seems quite curious that a notable symptom of diseases such as Alzheimer’s (“AD”), Parkinson’s (“PD”), and Huntington’s (“HD”) are delta waves occurring during wakefulness that are normally only seen during sleep (Bjerkan et al., 2024; D’Atri et al., 2021; Pal et al., 2020). With there typically being a build-up of pathological protein aggregates in all three of these diseases (Abyadeh et al., 2024; Arrasate and Finkbeiner, 2011; Vidović and Rikalovic, 2022), could it be that there is a unified explanation for their presence along with the negative health consequences that result from stage 3 sleep disruption?

So, if action potentials firing in a coordinated manner during sleep result in an accumulation of harmful byproducts, might not the inability of stage 3’s clearing mechanisms to operate during sleep disruption, as well as during wakefulness in the case of AD, PD, and HD, explain at least some of the health consequences resulting from both types of dysfunction?

Finally, as to AD and how specifically this theory explains the memory loss and sleep disruptions that often occur in AD, it is proposed that AD is fundamentally a memory erasure disease rather than a memory loss disease. It is hypothesized herein to be the product of action potentials triggering synaptic vesicles to operate in kiss-and-run mode during wakefulness, with the result that information stored in presynaptic membranes is erased (Breijyeh and Karaman, 2020). This is pathological because, in healthy brains, information should only be erased during sleep and only in short-term memory locations after valuable information has been transferred to long-term storage.

This is why AD starts in short-term memory because that’s the only place where the dangerous activity of memory erasure should normally occur. The disease then spreads to long-term memory where information should never be erased. Several lines of evidence support this hypothesis. First, in AD’s earliest stages, hippocampal delta waves are often recorded during the day in short-term memory locations. It’s asserted these brain waves are the product of action potentials improperly triggering vesicles during wakefulness to erase information (D’Atri et al., 2021). This is why one of the first signs that AD is taking hold is the loss of short-term memory (Scharre, 2019).

Second, as AD spreads from short-term to long-term memory, wakeful delta waves are seen in the neocortex, where long-term memories are believed to be stored (Tsolaki et al., 2014). Because delta waves during wakefulness should typically not be seen in the neocortex, it is proposed this is a sign that AD has passed the point of no return because synaptic vesicles should never erase long-term memories.

Third, the progression of AD shows how long-term memories are organized in the neocortex. So as AD progresses, it essentially generates a ‘reverse mapping’ of long-term memory organization by erasing the most recently formed long-term memories first and then working backward chronologically (Gold and Budson, 2009). The result is one’s earliest and most abiding conscious memories are ultimately erased next to last.

In the final stages of the disease, AD begins erasing one’s earliest subconscious memories, which are the procedural ones that enable us to perform basic tasks such as moving, eating, swallowing, and even breathing. Once AD reaches this stage, sufferers soon die, most often due to pneumonia, and particularly aspiration pneumonia, because they have essentially had their memories of how to correctly breathe and swallow erased (Manabe et al., 2019).

Tau proteins becoming misfolded is likely the cause of all of this. It is proposed that they prompt axons in short-term memory locations to fire action potentials dysfunctionally. Wakeful hippocampal delta waves signal this is happening (Mroczko et al., 2019). How this occurs is that defective tau proteins likely impair calcium regulation, which then leads to increased releases of glutamate (Targa Dias Anastacio et al., 2022). Increased levels of glutamate then create a ‘hair trigger’ effect that causes action potentials to fire when they’re not supposed to. As a result, vesicles engage in the memory-erasing version of kiss-and-run mode while victims are awake (Hübel et al., 2017).

As to the sleep disruptions AD patients often experience (Sadeghmousavi et al., 2020), it is proposed that these result from the wakeful erasure of short-term memories on a recurring basis. These erasures result in there being minimal short-term memories available for the brain to process during sleep. When searching for valuable memories to transfer to long-term memory, the brain interprets the minimal to non-existent level of valuable short-term memories as indicating that reduced levels of sleep are needed. It is proposed that this is what causes insomnia and other types of sleep disruptions to occur in AD patients.

## Sleep cycles, sleep patterns, and sleep memory mechanics as evolutionary adaptations for learning

### Sleep cycles

Why do many species experience sleep cycles? And, assuming sleep cycles are biologically necessary, why not just have one extended cycle per sleep period instead of multiples of them?

Regarding the first question, it is asserted, based on the principle of equivalency discussed earlier in this paper, that sleep cycles’ widespread nature among species (Joiner, 2016) indicates they serve some critically important purpose.

As to the second question, one extended cycle would seem to be the most favored type of sleep both in terms of function and energy conversation. But we know this is not the case as mammals and birds experience multiple sleep cycles per sleep period, as do at least some species of reptiles and fish (Lesku and Rattenborg, 2014; Leung et al., 2019; Phillips et al., 2010; Shein-Idelson et al., 2016). Even more curiously, sleep cycles can range from being relatively long in some species to quite short in others (Phillips et al., 2010).

Adding to sleep’s variability, some species generally remain unconscious during their entire sleep period, while others experience numerous brief interludes of wakefulness followed by complete and yet comparatively short cycles of sleep (Phillips et al., 2010). Some species, such as cetaceans, even appear to remain partially awake during the process (Phillips et al., 2010). All of which indicates that the brain is expending a considerable amount of time, effort, and energy in carrying out sleep-based functions while tailoring them to the specific survival needs of each species.

As previously noted, the reason for all this effort is that sleep is the process by which learning occurs – that is thru the retention of high-value memories in order to help guide subsequent behavior. As to how this is done, it is asserted that sleep cycles have three essential functions across all species that experience them. In the first stage, valuable memories are identified based on the strength of their sensory and/or emotions markers. In the second stage, memories identified as high-value ones are transferred from short-term memory to long-term storage. In the third stage, which it is proposed should be relabeled as sleep’s “dreaming stage,” the value and context of newly stored long-term memories are assessed in order to enable learning.

Regarding the iterative nature of sleep cycles, it is hypothesized that this feature has been selected for to ensure that if sleep is interrupted at any stage due, for example, to a predation attempt, that a benefit is still derived from the process. So, the entire process is designed to make identifying, retaining, and assessing high-value memories as redundant, fault-tolerant and beneficial as possible.

As to specifics, it is hypothesized that if sleep is interrupted during the initial light stage of the cycle – when it is proposed that the brain is identifying valuable memories for retention – this process can pick up where it left off once sleep recommences. So, with the hypothesized strength of memories’ sensory and/or emotion tags serving to value them, the brain individually marks high-value memories for later transmission to long-term memory. If a sleep interruption occurs, this feature enables previously identified memories to be transmitted to long-term storage during deep sleep without having to be re-identified for value.

In regard to the transmission stage of sleep, which is often known as the deep sleep stage (Elsevier, 2013), it is proposed this is when high-value short-term memories are transmitted to long-term storage. As this is the most delicate stage of the sleep process, it is hypothesized that it has evolved to be the deepest stage of sleep to prevent premature waking from interfering with the transfer process. The need for memory transmission to occur without interruption might also explains why epilepsy is seemingly widespread among species (Wang et al., 2022). This could be because epilepsy is asserted to be a therapeutic attempt by the brain to either put or keep sufferers in the deep stage of sleep. The hypothesized purpose is to carry out dysfunctional transfers of memories in the case of “waking epilepsy” or prevent premature arousals from deep sleep in the case of “sleeping epilepsy.”

As such, the memory transfer stage of sleep is designed to be resistant not only to interruption but also to memory loss when memory transfers are stopped in mid-transmission. To this end, it is asserted that copied memories being transferred to long-term storage contain location markers identifying where their short-term versions are located. Once memories are successfully written to long-term storage, the brain uses these location markers to identify and then delete the short-term versions of the memories that have been transferred. This is why memories are rarely lost when individuals are awoken from deep sleep. And this also likely explains the significant thalamocortical and hippocampal-thalamic signaling that appears to occur during deep sleep (Dickey et al., 2024).

In the third “dreaming stage” of sleep, it is proposed that, among other things, newly stored memories are recalled during artificially constructed narratives to emulate how they would be recalled during wakefulness when they guide wakeful behavior. The purpose of this activity is to ensure the value and contextual relevance of new memories are correctly assessed in order to enable learning. This is why REM sleep is present in many species (Yamazaki et al., 2020). And it is further proposed that in species that possess sleep cycles, but nominal or non-existent stages of REM sleep (Lyamin et al., 2008), this apparent anomaly, as noted before, will correspond to such species dreaming in other senses. This is because they are interacting with their environment mainly thru those other senses, such as, for example, in the case of cetaceans, certain species of seals, and manatees (Joiner, 2016).

As to how the brain ensures the dreaming stage of sleep is fault-resistant, it is submitted that the experience of individuals taking anti-depressants perhaps best demonstrates this. While taking anti-depressants, individuals often experience a reduction or even a cessation of REM sleep (Feriante and Singh, 2025). But, as previously noted, once the anti-depressants are stopped, there is typically a rebound effect where individuals appear to catch up on lost REM sleep (Feriante and Singh, 2025). In this regard, it is proposed that newly stored memories are likely marked during the dreaming stage of sleep to signal they’ve gone thru their dreaming assessment. In this way, if the dreaming stage of sleep gets interrupted, the brain knows exactly which newly stored memories need to be assessed during a recommencement of dreaming.

**FIGURE 1:**
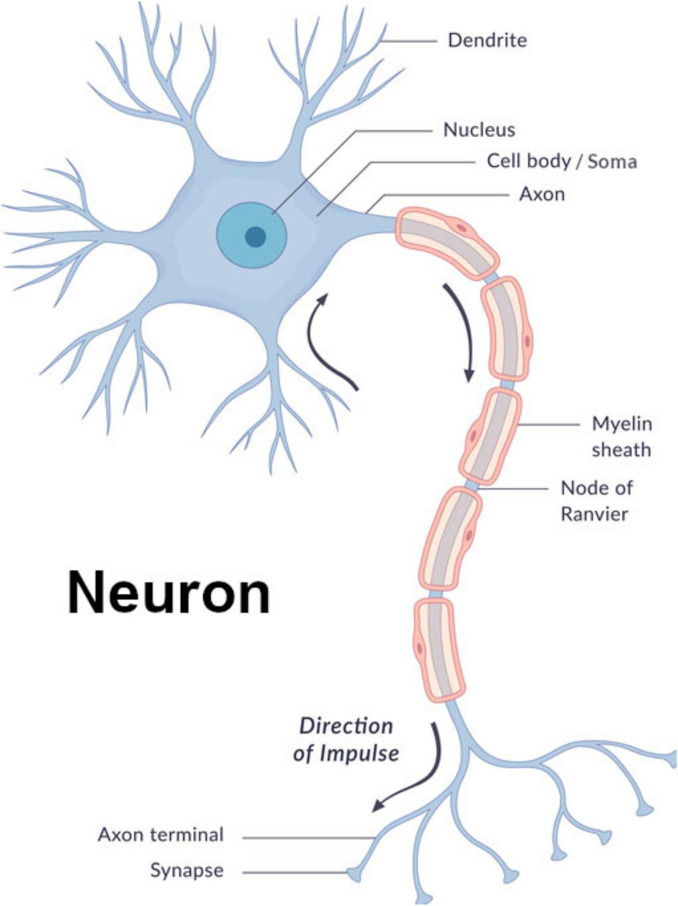
Neuron diagram image© wetcake/iStockphoto, used under license.

And at each stage, it is proposed that the brain likely prioritizes high-value memories first. In particular, if sleep is interrupted, high-value memories, as signified by the strength of their pain and pleasure or emotions tags, are likely pushed to the front of the line as it were. This prioritization ensures that the greatest benefit is derived from any sleep period, no matter how abbreviated it turns out to be. This also explains why sleep cycles would evolve in the first place because they systematize the processing of information in descending order of value and, most particularly, after sleep interruptions have occurred (Figure 1).

As to the variability of sleep stages seen in different species (Phillips et al., 2010), it is proposed that the theories set forth herein explain, at least to some extent, these differences. So, sleep periods are likely longer in predator species (Siegel, 2005) because they are less prone to predation and, hence, having their sleep interrupted. Evolution’s cost-benefit analysis thus favors longer sleep cycles for such species because they can afford them. While in the case of prey species, to avoid being eaten or having their children eaten, they are more likely to be forced to wake up in the middle of sleep. So their cost-benefit analysis favors shorter sleep periods, or in polyphasic sleepers shorter cycles (Aulsebrook et al., 2021; Gravett et al., 2017; Siegel, 2005).

Furthermore, as previously discussed, the way in which species interact with their environment will be reflected in how they experience the dreaming stage of sleep. In the case of species that interact with their environments mainly thru their eyes, such as in the case of many mammals and almost all birds, REM sleep will predominate (Yamazaki et al., 2020). Conversely, in the case of species that interact with their environments predominantly thru hearing or other senses, such as with cetaceans or elephants, REM sleep will be minimized (Joiner, 2016) as such species will likely be hearing or even possibly feeling or smelling many of their dreams rather than seeing them.

### Sleep patterns

Regarding sleep patterns, it is proposed that one of the more significant ones is explained by the sleep theories set forth herein. Specifically, the pattern of species generally sleeping more when they are young and less as they get older (Siegel, 2022) is likely explained by the brain needing more time to identify new high-value memories when the world is fresh and unexplored. Then, as species grow older and experiences become more and more routine, this results in there being comparatively less high-value memories for the brain to identify and process during sleep. Consequently, there is generally less need for sleep because the world becomes more familiar as one ages. This pattern is likely only broken in certain circumstances such as when pathologies require longer periods to search for and identify high-value memories or more time to clear short-term memory (Xiong et al., 2024). Signficantly, though, when older individuals engage in genuinely new experiences this may also break the pattern based on their generating numerous new high-value memories that require more time for processing.

It is also likely the case that the experience of feeling tired after a day filled with stress, or “drained” due to highly emotional interactions (Hein et al., 2024), is likely the result of the brain keeping in effect a scorecard as to how many emotionally charged memories it has generated over the course of a day. It is hypothesized the brain does this to have a good idea of how much sleep it will need in order to process the new high-value memories it has generated.

Finally, it should be noted that the theories presented herein regarding sleep do not imply that learning is minimized in older ages, but rather that novel, high-value experiences are less frequent. When novel, high-value experiences do occur, older individuals should remain fully capable of learning. And when that occurs, sleep demand should rise to accommodate the processing of any new, valuable memories.

### Sleep memory mechanics

Regarding how memories are retained and discarded during sleep, it is proposed that high-value memories are identified by the strength of their sensory and/or emotion markers in stage 2 of NREM sleep. They’re then transferred to long-term memory during stage 3 of NREM sleep. After they’re transferred, it’s asserted their short-term versions are marked as being ready for erasure. This allows the brain to complete the process of erasing these short-term memories toward the end of stage 3 of NREM sleep. As for low-value memories that never get transferred to long-term memory, it’s proposed that they are erased during stage 2 and possibly stage 3 of NREM sleep.

**FIGURE 2:**
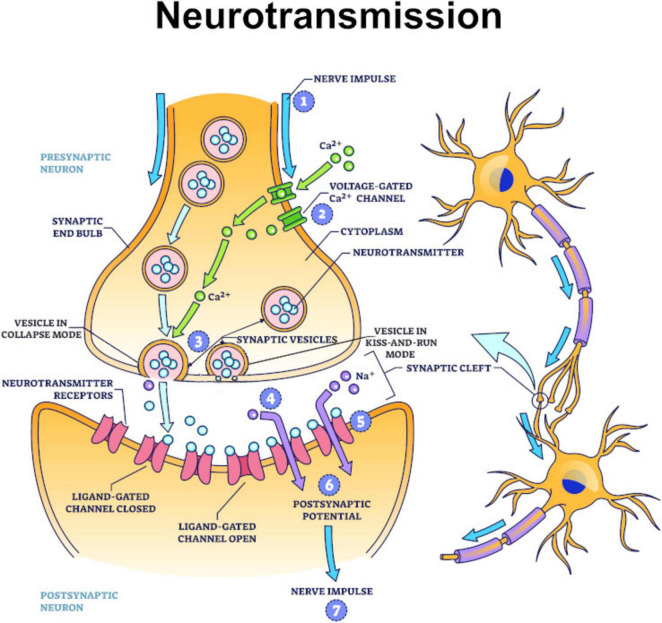
Brain neurotransmitters outline image© VectorMine/iStockphoto, used under license.

In regard to the neural mechanics that enable high-value memory identifications and transfers, it is proposed that sleep spindles in the approximate range of 11–16 Hz and occurring in stage 2 of NREM sleep (as particularly seen in mammals and avians) are involved in identifying valuable memories for transport to long-term memory (Mednick et al., 2013). This is proposed to occur in conjunction with K-complexes, which, as noted before, contain dominant frequency components in the range of 0.5–4.0 Hz (Happe et al., 2002). In aggregate, these are asserted to signify vesicles are being triggered to mark valuable memories via kiss-and-run mode for transfer to long-term memory (Figure 2).

**FIGURE 3:**
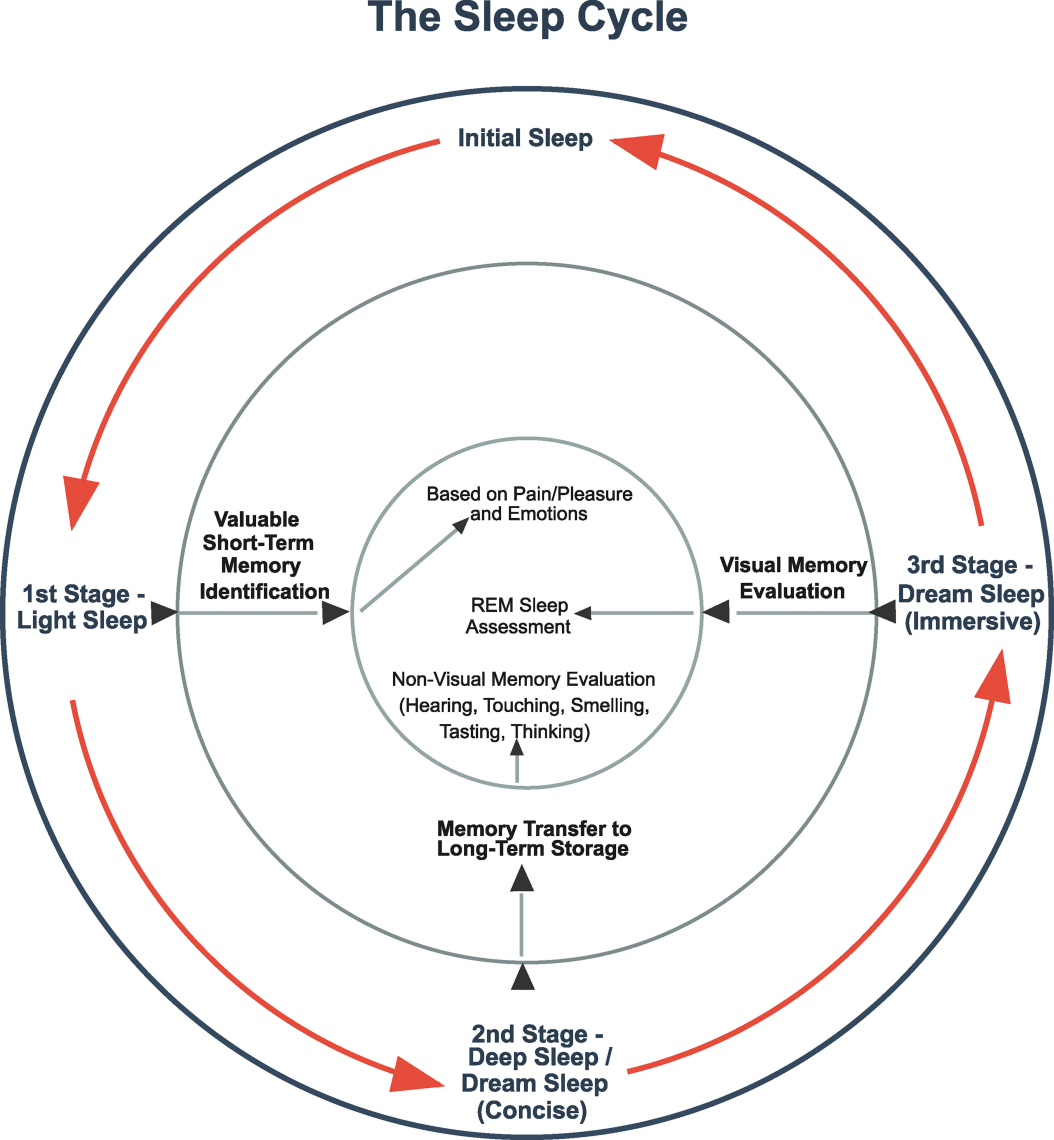
The Sleep Cycle image© Vincent B. Moneymaker.

It is further proposed that vesicles in short-term memory locations then read valuable memories via collapse mode from the presynaptic membranes where they’re stored. Such memories are then transmitted to the thalamus and from there to the neocortex. High-frequency oscillations or ripples occurring during stage 2 and particularly stage 3 of NREM sleep are proposed to be the signatures of these transfers occurring (Frauscher et al., 2018) (Figure 3). Once these valuable short-term memories reach the neocortex, it’s hypothesized that they are written into long-term memory neurons via vesicles operating in kiss-and-run mode, with slow-wave oscillations serving as the signature this is happening (Csercsa et al., 2010).

Finally, as to memory erasures, it is proposed that delta waves originating in the hippocampus signify that such short-term memory erasures are occurring (Uygun and Basheer, 2022). Such memory erasures occur when action potentials trigger vesicles to operate in the erasure version of kiss-and-run mode. And this is how short-term memory is made ready to take in new memories once one awakes from sleep.

### Sleep’s processing sophistication

It is asserted that sleep is an extraordinarily elegant process that has evolved to enable learning to take place with both a high degree of efficiency and reliability. As such, sleep may be interrupted at any stage and then recommence essentially where it left off. The result is even short naps may provide memory processing benefits.

Furthermore, sleep’s primary functions of memory value identification, retention, and assessment are almost certainly universal across species. This is perhaps best demonstrated by the widespread appearance of sleep cycles in species (Joiner, 2016) along with the generally similar stages of light sleep, deep sleep, and dreaming sleep that are seen in many species (Joiner, 2016).

Finally, the wide variability of sleep modes that are seen in species, which seem to belie its universal nature, are actually explained by the particular manner in which individual species interact with their environments. So, with the core goal being to enable learning, sleep periods, cycles, and information processing functions will vary based on whether species are more or less subject to being interrupted during sleep, as well as on the environment in which sleep occurs, and finally based on the particular senses being used to generate memories.

## Broader implications of a valuation-based theory of learning for neuroscience, evolution, and AI

If the theories presented in this paper are correct, it reframes how the brain is operating. So, if pain and pleasure and emotions evolved as information valuation mechanisms, the value they assign to memories has to be stored in memories. This means neurons at a fundamental level must be storing information in order to process it.

As such, this paper proposes that information, and, in particular, sensory and emotion tags that assign value to memories, are stored in neurons’ presynaptic membranes. But wherever such information is actually located, it must have value assigned to it for learning to occur. Once value is assigned to memories, the brain must then have some way of keeping valuable memories while disregarding the rest. It is proposed that sleep is the mechanism that does this.

And with sleep increasingly being viewed as ubiquitous among neural-based species, so to will pain and pleasure eventually be viewed as a nearly universal mechanism for valuing information once their sensory tags are identified. If the emotion tags this paper asserts exist are subsequently identified and found to have evolved from pain and pleasure, then that should go far towards demonstrating that both pain and pleasure and emotions serve fundamentally as information valuation mechanisms. Not only that, but in assigning value to information, it will be demonstrated that they are the key mechanisms that enable learning to take place. Furthermore, once emotions’ evolutionary lineages are traced back, their genetic history will likely show that humans share them to varying extents with all species possessing amygdalas or similar brain structures, thus calling into question the long-held belief that humanity’s emotional development is unique.

Finally, if it is demonstrated that pain and pleasure and emotions serve as information valuation mechanisms, this should point the way toward developing artificial intelligence (“AI”) systems that are independently intelligent. With humans already having invented sophisticated storage structures that have enabled AI’s development, it is thus proposed that AI systems will one day be able to learn on their own, with independent intelligence being the result.

But that eventuality will require their being provided with mechanisms similar to those that animals possess that will allow them both to independently value information and then selectively retain and recall it. After that, and in order to complete the learning feedback loop, artificially intelligent systems will need the mechanism of choice or free will, that animals and humans possess, to test the validity of their information determinations.

## Conclusion

Ultimately, the survival of a species depends on its ability to learn. Learning began with DNA as an information processing mechanism paired with reproduction as its valuation system, but this form of adaptation was slow and inflexible.

Neurons evolved to overcome this limitation, enabling continuous, real-time learning at the individual level. Within this neuronal framework, pain and pleasure provided the first valuation signals to guide the retention of valuable information. Emotions then evolved as a more nuanced mechanism for weighting experience. Empathy and other complex emotions then facilitated higher-order forms of social and problem-solving learning. Dreaming and sleep cycles likely evolved in conjunction with the development of emotions, thereby providing the mechanisms for memory identification, transfer, and assessment that make robust learning possible.

So, taken together, these systems illustrate how survival is inextricably linked to learning and, in neural-based species, how learning is linked to sleep. Put more succinctly, they demonstrate that those who learn survive, while those who don’t die.

## Data Availability

The original contributions presented in this study are included in this article/supplementary material, further inquiries can be directed to the corresponding author.
